# Inherited *CHST11/MIR3922* deletion is associated with a novel recessive syndrome presenting with skeletal malformation and malignant lymphoproliferative disease

**DOI:** 10.1002/mgg3.152

**Published:** 2015-05-10

**Authors:** Sameer S Chopra, Ignaty Leshchiner, Hatice Duzkale, Heather McLaughlin, Monica Giovanni, Chengsheng Zhang, Nathan Stitziel, Joyce Fingeroth, Robin M Joyce, Matthew Lebo, Heidi Rehm, Dana Vuzman, Richard Maas, Shamil R Sunyaev, Michael Murray, Christopher A Cassa

**Affiliations:** 1 Dana Farber Cancer Institute, Brigham and Women’s HospitalBoston, Massachusetts; 2 Broad Institute, Brigham and Women’s HospitalCambridge, Massachusetts; 3 Department of Medical Genetics, Yeditepe University School of MedicineIstanbul, Turkey; 4 Genetic Training Program, Harvard Medical SchoolBoston, Massachusetts; 5 Partners Healthcare Center for Personalized MedicineCambridge, Massachusetts; 6 Geisinger Genomic Medicine Center, Geisinger Medical CenterDanville, Pennsylvania; 7 The Jackson Laboratory for Genomic MedicineFarmington, Connecticut; 8 Cardiovascular Division, Washington University School of MedicineSt. Louis, Missouri; 9 University of Massachusetts Medical SchoolWorchester, Massachusetts; 10 Beth Israel Deaconess Medical CenterBoston, Massachusetts; 11 Division of Genetics, Department of Medicine, Brigham and Women’s Hospital, Harvard Medical SchoolBoston, Massachusetts

**Keywords:** *CHST11*, inherited lymphoproliferative disorder, malignant lymphoproliferative disorder, *MIR3922*, skeletal malformation

## Abstract

Glycosaminoglycans (GAGs) such as chondroitin are ubiquitous disaccharide carbohydrate chains that contribute to the formation and function of proteoglycans at the cell membrane and in the extracellular matrix. Although GAG-modifying enzymes are required for diverse cellular functions, the role of these proteins in human development and disease is less well understood. Here, we describe two sisters out of seven siblings affected by congenital limb malformation and malignant lymphoproliferative disease. Using Whole-Genome Sequencing (WGS), we identified in the proband deletion of a 55 kb region within chromosome 12q23 that encompasses part of *CHST11* (encoding chondroitin-4-sulfotransferase 1) and an embedded microRNA (*MIR3922*). The deletion was homozygous in the proband but not in each of three unaffected siblings. Genotyping data from the 1000 Genomes Project suggest that deletions inclusive of both *CHST11* and *MIR3922* are rare events. Given that *CHST11* deficiency causes severe chondrodysplasia in mice that is similar to human limb malformation, these results underscore the importance of chondroitin modification in normal skeletal development. Our findings also potentially reveal an unexpected role for *CHST11* and/or *MIR3922* as tumor suppressors whose disruption may contribute to malignant lymphoproliferative disease.

## Introduction

Glycosaminoglycans (GAGs) such as chondroitin and dermatan are a heterogeneous group of unbranched polysaccharides of variable length that consist of repeating disaccharides. With the exception of hyaluronic acid, GAGs are synthesized in the Golgi and then modified by enzymes including sulfotransferases. Chondroitin (the most prevalent GAG), heparin, dermatan, and keratin are all sulfated and subsequently appended to newly synthesized proteins via *O*- or *N*-linked glycosylation to form mature proteoglycans. Proteoglycans function at the cell membrane or as secreted components of the extracellular matrix (Theocharis et al. 2010).

Although GAG-modifying genes such as sulfotransferases are required for normal GAG and proteoglycan synthesis, the role of these genes in human development and disease is less well understood. Here, we report a previously undescribed syndrome whose cardinal features include bilateral bony deformities of upper and lower extremities and susceptibility to malignant lymphoproliferative disease. The phenotype was present in two of seven siblings but in neither parent, suggestive of a genetic disorder with an autosomal recessive mode of inheritance. Whole-Genome Sequencing (WGS) of the proband revealed a biallelic 55 kb deletion at chromosome 12q23 spanning part of the locus for *CHST11* and including an embedded *MIR3922*. Nullizygosity for the deletion was observed in the proband and not in three unaffected living siblings who subsequently underwent testing. When considered together with the known role for *CHST11* in murine cartilage growth plate formation during development, our findings strongly suggest that chondroitin sulfation is required for normal development of the bony skeleton in humans. Our findings also support the hypothesis that *CHST11* and/or *MIR3922*, a microRNA whose function has not previously been described, may act as tumor suppressors in the lymphocyte lineage.

### Clinical history

**Figure 1:**
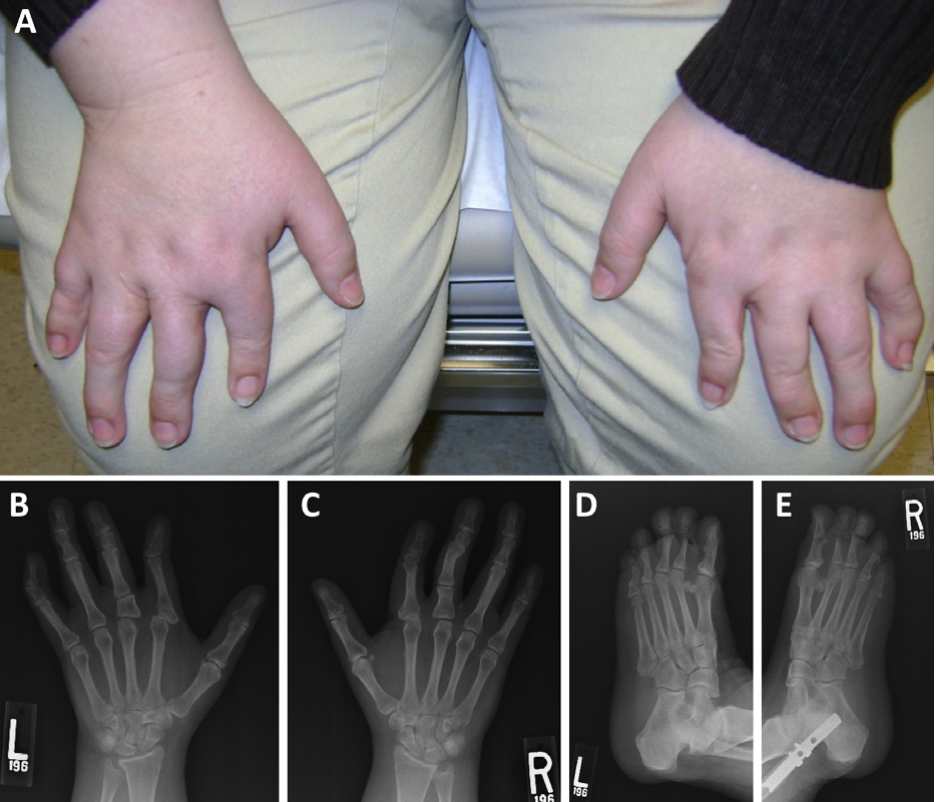
(A) At present, the hands of the proband are notable for malformed digits. (B and C) X-rays of the hands at present. *See text for detailed description*. (D and E) X-rays of the feet at present. *See text for detailed description*.

The proband is a 46-year-old woman who initially presented to the adult genetics clinic at Brigham and Women’s Hospital in 2005 for evaluation of a personal and family history of congenital hand/foot malformations and peripheral T-cell lymphoma. At 61 in. in height (10th percentile, CDC growth curves), she is shorter than all of her first-degree relatives except one similarly affected sister. She was born with bony abnormalities of the fingers and toes bilaterally, for which she underwent multiple surgical repairs in early childhood. On clinical exam, her fingers demonstrated brachydactyly with disproportionately short index fingers and either valgus or varus deformity at many of the interphalangeal (IP) joints (Fig.1A). Similar findings were observed in the toes. X-ray images were taken of the hands and feet, with multiple notable findings. On the left hand, the index finger displayed a long proximal phalanx, a fused distal phalanx and subluxation of the index metacarpophalangeal (MCP) joint with remodeling of the bony surfaces; the third digit had four phalanges and a horizontal articulation at the base of the proximal phalanx; the fourth and fifth fingers each had long proximal phalanges; and the fifth finger middle phalanx had a curved articulation distally (Fig.1B). The metacarpals appeared more normal with the exception of a hypoplastic index metacarpal. The thumbs and wrist bones appeared normal. Similar findings were observed in radiographs of the right hand (Fig.1C).

In the left foot, plain films revealed fusion across the great toe IP joint; small middle phalanges of the second and third toes; and only two phalanges of the fourth toe with a well-defined lucency at the base of the proximal phalanx of the fourth toe, thought to be a separate ossicle (Fig.1D). The fifth toe showed a faint lucency with sclerotic margins across the proximal phalanx. The metatarsals demonstrated no abnormalities. The right foot displayed similar abnormalities (Fig.1E).

The proband’s medical history was further complicated at 44 years of age, when she developed several weeks of fever, flu-like symptoms, and pulmonary infiltrates unresponsive to antibiotics that culminated in respiratory failure and mechanical ventilation. Extensive testing for an infectious etiology was negative and she was diagnosed with acute respiratory distress syndrome (ARDS) of uncertain etiology. Her symptoms ultimately improved with high-dose steroids but recurred when steroids were discontinued. Surgical lung biopsy revealed an atypical lymphoid infiltrate consisting primarily of CD3+ T cells but with a minor component of CD20+ B cells. Immunoglobulin heavy chain polymerase chain reaction (PCR) did not identify any evidence of B-cell clonality. The histologic pattern of an angiocentric lymphoid infiltrate was suggestive of lymphomatoid granulomatosis, an Epstein–Barr virus (EBV)-associated lymphoproliferative disorder. However, additional evidence supporting this diagnosis including atypical lymphoid cells reactive for B-cell antigens or EBV-associated mRNAs was not identified. Bone marrow biopsy was unremarkable. The patient was restarted on antibiotics and steroids and improved clinically. Her lung biopsy was reviewed at the National Cancer Institute and the overall impression was that of a reactive process, possibly an organizing pneumonia, rather than a primary lymphoproliferative disorder such as lymphomatoid granulomatosis, although the latter could not be definitively ruled out. The patient’s condition ultimately went without diagnosis.

She subsequently underwent a second lung biopsy, again revealing nodular lymphoid infiltrates of primarily CD3+ T cells with scattered CD20+ B cells and CD68+ histiocytes. PCR for rearranged immunoglobulin heavy chain and T-cell receptor (TCR) genes did not demonstrate any evidence of B- or T-cell clonality, respectively. Flow cytometry of peripheral blood lymphocytes revealed no abnormalities. Overall, definitive diagnostic features of a T- or B-cell lymphoma were not observed. Based on clinical suspicion of malignancy, the patient was treated with rituximab followed by four cycles of chemotherapy with cyclophosphamide, doxorubicin, vincristine, and prednisone (CHOP). The patient’s clinical symptoms improved in concert with partial regression of her pulmonary infiltrates. New wedge biopsies of the right middle and upper lobes revealed a nodular, angiocentric and bronchocentric atypical T-cell lymphoid infiltrate extending into intra-alveolar spaces. The infiltrate was composed of medium size and large atypical lymphoid cells admixed with small lymphocytes and histiocytes. Immunohistochemical studies revealed that the majority of large atypical lymphoid cells stained positively for the T-cell markers CD3, CD4, and CD8 (minority). CD5 expression was absent but for a small number of cells. The infiltrating T cells were also positive for CD43 and TIA-1 in a granular cytoplasmic pattern. EBV LMP-1 was negative, as were B-cell markers CD20 and CD79a and the natural killer cell marker CD56. Additional negative markers included CD30, CD15, CD34, MPO, and CD1a. Ki-67 stained approximately 20–30% of cells. EBV mRNA by in situ hybridization was also negative. On this occasion, TCR gamma gene rearrangement studies revealed evidence of clonality. The patient’s progressive clinical course, the cytologically atypical T-cell infiltrate, the immunophenotypic aberrancy (absence of CD5), the lack of EBV mRNA, and molecular evidence of clonality with an identical pattern observed at two different anatomical sites (right middle lobe, right upper lobe) were most consistent with a diagnosis of peripheral T-cell lymphoma. She received additional chemotherapy prior to undergoing a matched unrelated allogeneic bone marrow transplant from a male donor. Her clinical course has been complicated only by grade 1 graft versus host disease and several non-life-threatening infections. She continues to have no evidence of disease on periodic restaging positron emission tomography–computed tomography (PET-CT) scans.

The proband’s family history is most notable for an older sister with similar congenital malformation of the hands/feet who succumbed to complications of an unnamed lymphoproliferative disorder in her early 20s. At a height of sixty inches, the deceased sister was also shorter than all other first-degree relatives. A review of her medical records from the 1980s revealed that she presented at 17 years of age with abdominal pain, nausea, and vomiting, subsequently found to be caused by a small bowel obstruction complicated by perforation and pneumoperitoneum. She also described a several week history of skin lesions of uncertain origin, fever, shortness of breath, and tachypnea. Radiographic imaging of the lungs revealed diffuse bilateral nodular infiltrates. Management of the small bowel perforation ultimately included partial ileal resection, right-sided colectomy, and jejunostomy. Per report, pathologic evaluation of the resected small bowel revealed dense lymphocytic infiltrates. Skin biopsy similarly revealed a lymphocytic infiltrate. Biopsy of the lung was notable for an angiocentric lymphocytic infiltrate resembling lymphomatoid granulomatosis, the same EBV-related condition initially suspected to underlie the proband’s pulmonary lymphoid infiltrates. A bone marrow biopsy was reportedly normal on two occasions. No additional molecular diagnostic testing was available and/or performed at the time. She was treated with cyclophosphamide, azathioprine, and prednisone with resolution of both pulmonary infiltrates and cutaneous manifestations. However, her course was subsequently complicated by new-onset seizures. Computed tomography (CT) imaging suggested involvement of the central nervous system (CNS) with the lymphoproliferative disorder. A brain biopsy was performed and the pathology was consistent with malignant lymphoma of the CNS, not otherwise specified. She ultimately died from complications of her disease at age 22.

**Figure 2:**
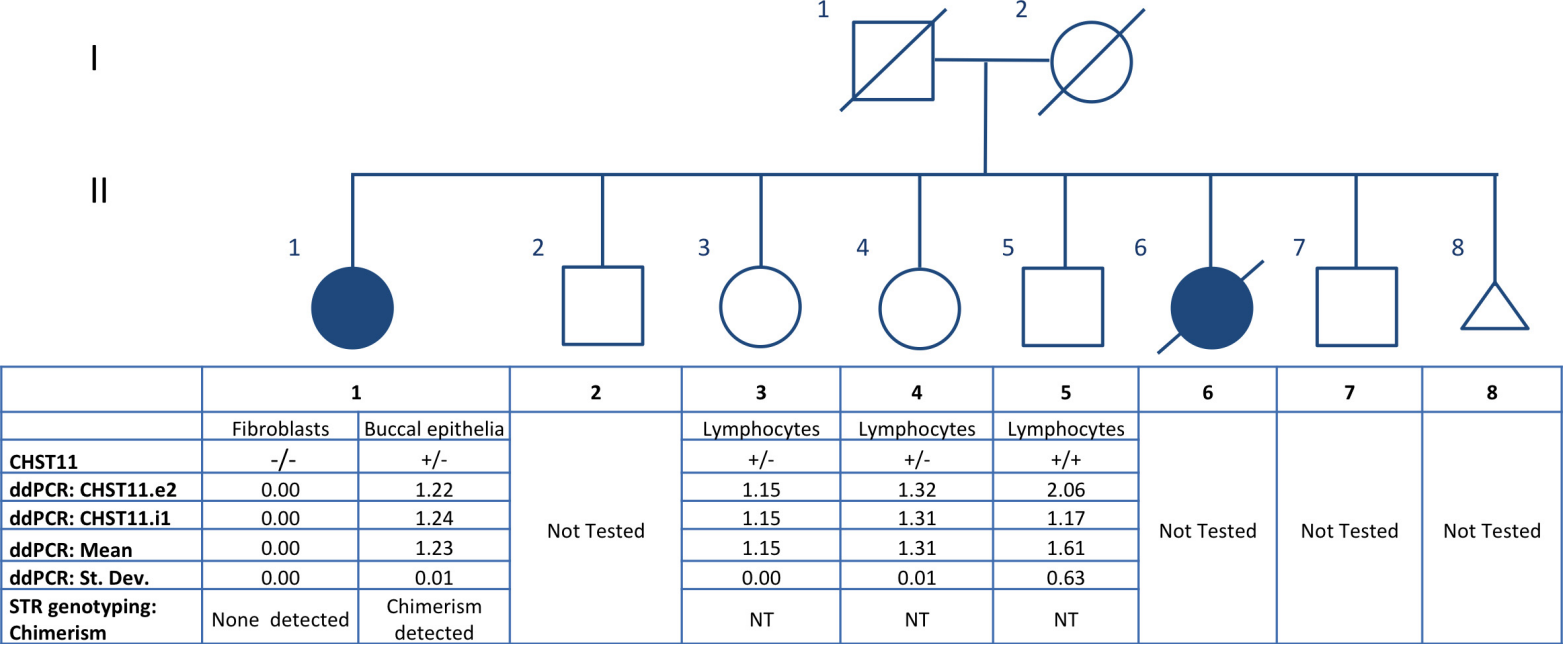
The pedigree demonstrates a large sibship with two affected individuals, one deceased. The table below the pedigree indicates the genotyping results for the proband and the three siblings who underwent testing. The data include number of *CHST11* deletion alleles (homozygous null, heterozygous, or homozygous wild type) as well as results of droplet digital PCR (exon 2, intron 1), including mean and standard deviation. STR genotyping results (for chimerism) are also indicated.

The proband and her deceased sister have five unaffected living siblings (Fig.2). No other individuals in the extended family are reported to have congenital abnormalities of the hands or feet. With the exception of the proband’s father, who was diagnosed with chronic lymphocytic leukemia (CLL) at age 59 and died at age 64, no other individuals have developed any lymphoproliferative disorders. The proband’s parents were both immigrants to the US from the same region of Ireland, so the possibility of distant consanguinity cannot be ruled out. The family history is most consistent with a rare monogenic disorder of autosomal recessive inheritance.

A search of online databases including OMIM and PubMed did not identify any named syndrome associated with both skeletal dysplasia and any type of lymphoproliferative disorder. Therefore, no specific candidate gene rose to a level of suspicion to warrant clinical testing. The results of chromosome breakage analysis with diepoxybutane (DEB) were normal and therefore did not support a diagnosis of Fanconi anemia (FA). Array-based comparative genomic hybridization using a custom-designed platform with 180,880 probes throughout the human genome did not reveal any clinically significant genomic gains or losses. Therefore, we performed WGS on genetic material obtained from the proband to identify rare homozygous variants that could potentially explain the phenotype in this family.

## Methods

### Consent

Consent to perform WGS under Clinical Laboratory Improvement Amendments (CLIA) conditions was obtained under protocols approved by the Institutional Review Board (IRB) of Partners HealthCare, Inc. Explicit permission to publish the findings contained herein was obtained from the proband.

### Skin biopsy and fibroblast culture

Due to the patient’s prior allogeneic bone marrow transplant, which resulted in reconstitution of the entire hematopoietic system by donor cells, we isolated and sequenced genomic DNA from cultured skin fibroblasts rather than from circulating (donor) hematopoietic cells (Valencia et al. 2014). The skin biopsy was rinsed, minced, and then cultured in P-60 dishes containing 5 mL complete minimal essential media medium with 10% fetal bovine serum (FBS), penicillin and streptomycin (100 units/mL), and gentamicin (50 *μ*g/mL). The dishes were incubated at 37°C with 5% CO_2_ and examined daily for cell growth. When the cells became 90–100% confluent in culture, genomic DNA was isolated from fibroblasts using the Gentra Puregene Cell Kit (QIAGEN, Venlo, Netherlands) and subsequently used for the WGS sequencing.

### Whole-genome sequencing

WGS was performed by the Illumina Clinical Services Laboratory (Illumina, Inc; San Diego, CA). Briefly, genomic DNA was randomly fragmented and then sequenced using 50 nt paired-end reads on an Illumina HiSeq 2000 Sequencer at an average depth of 30× coverage (Bentley et al. 2008). Resulting BAM files were converted to FASTQ format and realigned to the human reference sequence [University of California Santa Cruz (UCSC) hg19/GRCh37 assembly] using the Burroughs-Wheeler Alignment tool (BWA) (Li and Durbin 2009) in paired-end mode. Duplicated reads resulting from PCR overamplification or optical duplication were flagged and discarded by the MarkDuplicates module of Picard tools (version 1.67). Targeted local realignment was then performed near known short insertions and deletions (indels) and by base quality recalibration using the Genome Analysis Toolkit (GATK, version v2.1-8-g5efb575) (McKenna et al. 2010; DePristo et al. 2011). Single-nucleotide variants (SNVs) and indels were identified using batch calling with a set of unrelated individuals by using the Unified Genotyper module in the GATK in multisample calling mode. Suspected sequencing artifacts that were present across the samples of several unrelated individuals were removed. Variant quality score recalibration was performed using the GATK to identify a set of high-confidence candidate variants. The functional consequence of each variant was predicted using Variant Effect Predictor (Release 71) (Yourshaw et al. 2014). Variants not found in dbSNP (Single Nucleotide Polymorphism Database) build 135 (Sherry et al. 2001), in the 1000 Genomes Project (1KG) and the Exome Sequencing Project (ESP, *N* = 6503) data sets were considered for analysis (NIH 2012) (1000 Genomes Project FTP Server 2012). A recessive inheritance scenario was most probable in this case, so a stringent threshold of 0.1% population frequency in ESP and 1KG was used, resulting in rare or private candidate variants. Homozygous and compound heterozygous sequence variants were evaluated. Structural variants (SV) were identified using a panel of WGS from 946 unrelated individuals by comparing the average coverage for controls and the proband’s sample over the same exons. The resulting SV variants were genotyped across individuals from the 1000 Genomes Project to assess the SV’s population frequency, and SVs with minor allele frequency over 1% were filtered using GenomeSTRiP (Handsaker et al. 2011).

### Droplet digital PCR (ddPCR) assay

The homozygous deletion of *CHST11* and *MIR3922* identified by WGS of DNA from the proband’s cultured fibroblast cells was confirmed by testing the proband’s DNA from both her cultured fibroblasts and buccal epithelia simultaneously by ddPCR. TaqMan assays recognizing target genes were designed for a region on intron 1 (also overlapping *MIR3922*), exon 2 of *CHST11*, along with reference genes *RPP30* and *AP3B1* (sequences and other information can be found in Table S1). Each assay employed forward and reverse primers and an MGB-TaqMan probe labeled with either FAM- or VIC- dyes custom-designed using PrimerExpress software (Life Technologies, Waltham, MA) and synthesized by Life Technologies. Assays were designed specifically to exclude a *MseI* cutting site within the amplicon since *MseI* was used to fragment DNA into smaller pieces and to avoid copy number polymorphisms in the general population in the Database of Genomic Variants (MacDonald et al. 2013). The detailed workflow for ddPCR has been described previously (Hindson et al. 2011). Raw data analysis was automatically performed by QuantaSoft software (Bio-Rad, Hercules, CA), followed by manual normalization of the raw copy number values using a correction factor to yield a copy number of 2.0 to the *RPP30* reference gene.

### Identity testing

To confirm that deletion of *CHST11* and *MIR3922* was present in the constitutional genome of the proband and not an artifact induced by culturing skin fibroblasts, we performed genotyping of highly polymorphic short tandem repeats (STRs) using genomic DNA from the proband’s buccal epithelia and cultured fibroblast cells. A multiplex PCR reaction was performed for 16 STR loci using the commercially available AmpFlSTR® IdentifilerTM PCR Amplification kit (Applied Biosystems; Carlsbad, CA), following the manufacturer’s instructions. Resulting STR amplicons were separated and visualized using an ABI Prism® (Waltham, MA) Genetic Analyzer. STR alleles were scored using GeneMarker software v1.5 (Softgenetics, State College, PA).

## Results

Given the recessive mode of inheritance observed for the phenotype in this family, we searched the proband’s WGS data for genes harboring rare biallelic single-nucleotide and compound heterozygous variants (Table1). We identified compound heterozygous variants in *MBD3L5* and *PRND*. A homozygous missense variant in *EPHA10* and a homozygous variant in *OLA1* 3′UTR were also identified. While the variants in these four genes appeared to be technically valid, there was insufficient evidence based on known expression and/or biological function to link these variants to the observed phenotype.

**Table 1:** Other variants identified during whole-genome sequencing

Gene	Chromosome	Coordinate	Ref	Alt	Protein Chg	Notes
Compound heterozygous variants						
MBD3L5	chr19	7032665	A	G	R129R	Synonymous
MBD3L5	chr19	7032688	T	G	I137S	Missense
PRND	chr20	4706780	C	T	UTR 3′	Possible artifact
PRND	chr20	4706782	A	T	UTR 3′	Possible artifact
SIRT6	chr19	4174745	C	A	A241S	Missense
SIRT6	chr19	4174758	G	C	N236K	Missense
Homozygous alternate variants						
EPHA10	chr1	38218738	G	C	C389W	Missense
OLA1	chr2	175113308	G	A	UTR 3′	UTR 3′

Other compound heterozygous and homozygous alternative variants were identified during whole-genome sequence interpretation. These variants have been ruled out as causal in this case using our interpretation pipeline.

We next examined the proband’s WGS data for homozygous deletions too small to be detected by array-based comparative genomic hybridization. Three such deletions were identified: chr12:104,948,000-105,003,000 (55 kb), chr19:55,253,424-55,295,014 (41.5 kb), and chr19:54,801,000-54,807,500 (6.5 kb). The first deletion included exon 2 of the gene *CHST11* as well as the embedded microRNA *MIR3922*. By computational prediction, *MIR3922* may target *ZNF585A* (Zinc Finger Protein 585A [19q13.12]), *RNF214* (Ring Finger Protein 214 [11q23.3]), *RAB10* (RAS-Related Protein RAB-10 [2p23.3]) and/or *ERBB2* (erythroblastic leukemia viral oncogene homolog 2) (Maragkakis et al. 2009). The two deletions of chromosome 19 included the genes *KIR2DL3*, *KIR2DL1,* and *LILRA3*.

**Figure 3:**
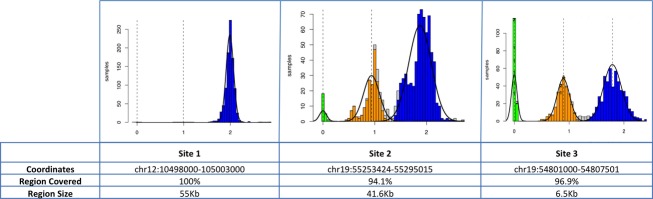
Three homozygous deletions were identified in genomic DNA from the proband. The population frequency of these deletions was determined using genotyping data generated by Genome STRiP in the 1000 genomes cohort. As shown in the histograms, the two deletions on chromosome 19 have many individuals with deletions in the 1KG samples (>1%), while the deletion on chromosome 12 that includes *CHST11* and *MIR3922* appears to be a rare variant, with no deletions observed in the 1KG project.

**Figure 4:**
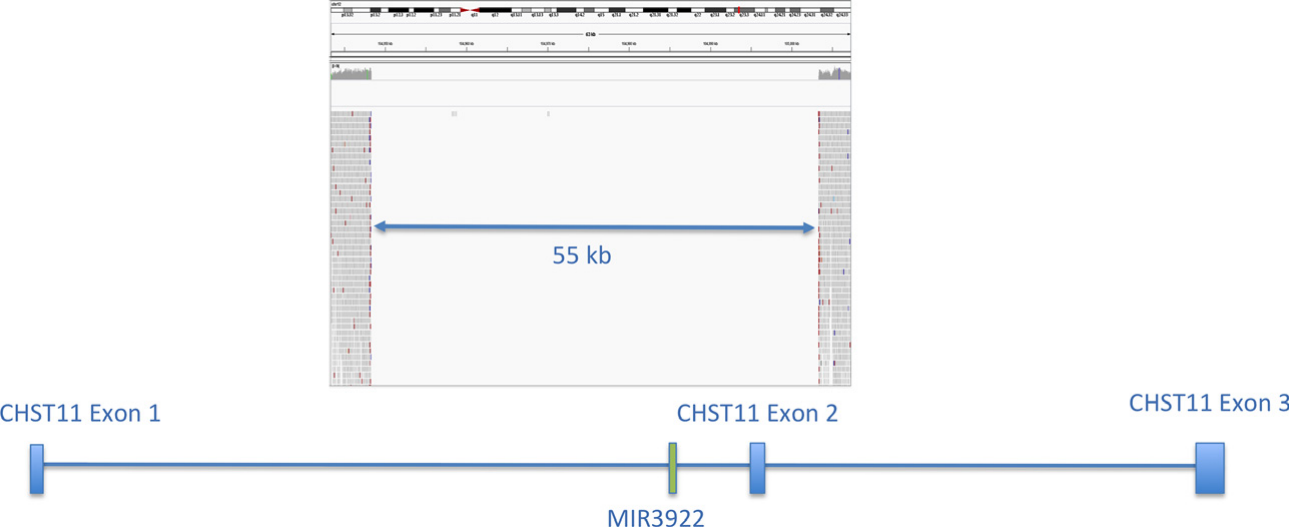
There are very few observed sequence reads in the region between chr12:104,948,000-105,005,000, providing much more precise resolution on the boundaries of the deletion.

The population frequency of these homozygous deletions was determined using genotyping data generated by Genome STRiP in the 1KG cohort (Fig.3). The two deletions on chromosome 19 have many individuals with deletions in the 1KG samples (>1%), while the deletion on chromosome 12 that includes CHST11 and MIR3922 appears to be a rare variant, with no deletions observed in the 1KG project. The parents of the proband were born in the same region in Ireland, suggesting that the rare homozygous deletion could be a part of a region where there is identity by descent (IBD). Indeed, a potential IBD region of approximately 180 kb overlaps the entire deletion, spanning chr12:104,920,000-105,100,000 (Fig.4).

To rule out the possibility of culture artifact causing homozygous deletion of *CHST11* and *MIR3922*, saliva was obtained from the proband and genomic DNA was isolated from buccal epithelial cells. Droplet digital PCR (ddPCR) was performed to analyze the copy number of *CHST11* and *MIR3922* alleles. The average *CHST11* copy number was confirmed to be 0 in the fibroblast cells and 1.23 in buccal epithelial cells (*σ* = 0.01). This unexpected result suggested the possibility of genomic heterogeneity secondary to a mixed population of host and donor-derived cells/genomes or due to chimerism from variable fusion of *CHST11*-null host DNA with *CHST11*-diploid donor genomic DNA (Ferrand et al. 2010). To evaluate for genomic heterogeneity, we performed identity testing by microsatellite genotyping and compared the results from genomic material isolated from fibroblasts to those from buccal epithelium (Table S2). Analysis of microsatellite markers in genomic DNA isolated from fibroblast culture was consistent with origin from a single individual. The same analysis of genomic DNA isolated from buccal epithelium, however, demonstrated evidence of chimerism at 13/16 loci and the presence of a Y chromosome marker within the female proband, confirming the presence of genetic material originating from the male bone marrow donor. When chimerism was calculated on 10 of 13 informative loci the mean value was found to be 62%, consistent with 1.23 alleles contributed by the *CHST11*-diploid donor as determined by ddPCR. When taken together, these findings strongly suggest that homozygous deletion of *CHST11* and *MIR3922* in skin-derived fibroblasts is representative of the proband’s germline and not the result of a culture artifact.

To determine whether deletion of *CHST11* and/or *MIR3922* is likely to underlie the combined phenotype of skeletal malformation and T-cell lymphoma in this family, we attempted to obtain biological material from the proband’s affected (deceased) sister but were ultimately unsuccessful. Instead, we analyzed the copy number of *CHST11* and *MIR3922* in the proband’s unaffected siblings. A ddPCR analysis performed for the *CHST11* and *MIR3922* loci did not reveal homozygous deletion of *CHST11* and *MIR3922* in any of the three unaffected siblings who were tested (Fig.2).

To determine whether somatic copy number changes or mutations in *CHST11* and/or *MIR3922* are commonly found in sporadic hematologic malignancies, we searched the Broad TumorPortal and Catalog of Somatic Mutations in Cancer (COSMIC) databases (Koboldt et al. 2012). No copy number variants in *CHST11 (and by extension MIR3922)* were reported for the available sequences from hematopoietic or lymphoid tumor samples. However, somatic copy number variants in *CHST11* were identified in several solid tumor samples. In the COSMIC database (accessed December 2013), 511 samples representing 8 of 27 solid tumor types were found to harbor copy number loss in the *CHST11* locus. *CHST11* copy number loss was most common in ovarian cancer (31.2% of samples). In addition, 28 and 33 nonsynonymous SNVs in *CHST11* are reported in the Broad TumorPortal and COSMIC, respectively. Notable variants include missense substitutions in diffuse large B-cell lymphoma (p.P99Q) and CLL (p.R42L), a frameshift (p.F31 fs) in clear cell renal carcinoma, and nonsense variants in esophageal cancer (p.Q329*), lung adenocarcinoma (p.E325*), and liver cancer (p.E239*). The functional significance of these variants is presently unknown. No variant data are reported for *MIR3922*. Of note, a somatic translocation involving *CHST11* (t(12;14)(q23;q32)) was previously identified in a patient with B-cell chronic lymphocytic leukemia (B-CLL) (Schmidt et al. 2004).

## Discussion

In this study, we identified biallelic deletion of *CHST11/MIR3922* in the proband as the putative cause of an unnamed autosomal recessive syndrome presenting with congenital malformations of the digits of the upper and lower limbs and malignant lymphoproliferative disease. Homozygosity for the deletion allele was not observed in three unaffected living siblings who were available for testing. Unfortunately, biological material from the deceased affected sister was not available. Although the proband’s clonal lymphoproliferative disorder was immunophenotypically classified as T-cell lymphoma, the lack of molecular classification of her affected sister’s lymphoma in the 1980s prevents us from definitively associating *CHST11/MIR3922* deletion specifically with abnormalities of the T-cell lineage.

The rare homozygous deletion identified in the proband is unlikely to be an artifact. While ddPCR of buccal epithelium from saliva demonstrated hemizygosity for the *CHST11/MIR3922* locus, extensive chimerism at 13 of 16 informative STR markers (including a donor Y chromosome) was detected in saliva from our female proband. This suggests the contribution of donor cell-derived genomic DNA to the proband’s gastrointestinal tract epithelium, as has been previously described (Ferrand et al. 2010; Rennert et al. 2013).

Our analysis of existing genomic databases suggests that the *CHST11* locus is relatively intolerant of deletions and loss-of-function variants. Although a study using genome-wide BAC aCGH relative expression levels across 95 samples reported a hemizygous deletion near the beginning of the *CHST11* gene with 4% frequency (Wong et al. 2006), no deletion events at this locus were observed using higher quality whole-genome sequence data across 946 samples in the 1KG data set. The same study also reported a complex structural rearrangement encompassing *CHST11* and *MIR3922*, but similar rearrangements were neither observed in the 1KG data set nor would necessarily preclude wild-type expression and function. It seems implausible to observe 5 true SVs in 95 samples but no such events in 946 samples analyzed using higher quality data and more sensitive methods of detection. Therefore, we believe that *CHST11*/*MIR3922* SVs are rare. In more than 63,000 exomes cataloged at the Broad Institute, no homozygous loss-of-function variants were observed in either *CHST11* or *MIR3922,* further supporting the hypothesis that biallelic deletion of the locus best accounts for the proband’s phenotype (exome data available at http://exac.broadinstitute.org/gene/ENSG00000171310).

Our review of the literature suggests that the combined phenotype of skeletal dysplasia and malignant lymphoproliferative disease is rare. A single case report describes an infant with short-limbed skeletal dysplasia and severe combined immunodeficiency who developed EBV-associated B-cell lymphoma at 10 months of age (van den Berg et al. 1997). Another report describes two sisters with skeletal dysplasia and Hodgkin’s lymphoma arising in late adolescence (Gokhale et al. 1995). However, no specific genetic etiology was identified to account for the clinical presentation.

Notably, the phenotype observed in our proband resembles other rare Mendelian disorders that combine skeletal anomalies with specific hematopoietic derangements. The recessive disorder thrombocytopenia-absent radius (TAR) syndrome is characterized by low platelets and anomalies of the radius and may also present with eosinophilia, anemia, various other skeletal abnormalities, and disturbances of other systems (skin, cardiovascular, neurologic). TAR has been associated with genetic variants in the gene *RBM8A*, a protein that serves as one component of the multiprotein exon junction complex bound to mRNA at splice junctions. Fanconi anemia is a genetically heterogeneous recessive disorder that presents with pancytopenia (or isolated cytopenias) and skeletal anomalies such as radial aplasia and thumb aplasia or hypoplasia, in addition to disturbances of several additional systems (genitourinary, skin, cardiac, neurologic). The common molecular defect shared by all FA complementation groups is in DNA repair, resulting in hypersensitivity to DNA cross-linking agents.

Chondroitin sulfate is the most common proteoglycan in cartilage and is present on the surface of cells in many tissue types as well as in the extracellular matrix. Chondroitin is an unbranched GAG that consists of alternating sugars of glucuronic acid and N-acetylgalactosamine (GalNAc). Sulfate moieties added at position 4 or 6 of GalNAc residues alter the physiochemical properties of chondroitin and chondroitin glycoconjugates. The *CHST11* gene encodes a chondroitin 4-sulfotransferase localized in the Golgi apparatus that catalyzes the transfer of sulfate to position 4 of GalNAc in chondroitin. *CHST11* is one of several known carbohydrate sulfotransferases, sharing four regions of homology including 5′ and 3′ phosphosulfate binding sites and two C-terminal alpha helical domains. The gene *CHST3*, for example, encodes a chondroitin 6-sulfotransferase that catalyzes the transfer of sulfate to position 6 of GalNAc in chondroitin.

The association of germline variation in *CHST11* with a clinical phenotype underscores the importance of carbohydrate sulfotransferase function in human biology. Biallelic loss-of-function mutations in genes encoding other carbohydrate sulfotransferases have been previously described in rare syndromes with autosomal recessive inheritance. Homozygous or compound heterozygous mutations in *CHST3*, encoding a chondroitin 6-sulfotransferase, are associated with recessive spondyloepiphyseal dysplasia and congenital joint dislocations (OMIM 143095) (Thiele et al. 2004; Unger et al. 2010). Inactivating mutations and deletions of *CHST6*, encoding a keratan 6-sulfotransferase expressed in the human cornea, have been associated with macular corneal dystrophy types I and II (Akama et al. 2000; Aldave et al. 2004). Loss-of-function mutations in *CHST14*, a dermatan 4-sulfotransferase, have been associated with Ehlers-Danlos syndrome musculocontractural type 1 (OMIM 601776) (Dündar et al. 2009).

The digital malformations exhibited by our proband and reportedly present in her affected sister suggest an important role for *CHST11* in normal bone development. Differentiation of chondrocytes is responsible for development of a cartilage model of future bone, and chondrodysplasias in mice are known to be associated with bone and joint abnormalities (McLean and Olsen 2001). In mice, *CHST11* is expressed in the apical ectodermal ridge of developing limbs and subsequently in the cartilage growth plate during embryogenesis (Klüppel et al. 2002). Mice homozygous for a loss-of-function mutation in the murine *CHST11* homolog die within hours of birth and exhibit severe chondrodysplasia characterized by disorganized cartilage growth plates (Klüppel et al. 2005). This phenotype is associated with imbalance of chondroitin sulfation, mislocalization of chondroitin sulfate in growth plates, upregulation of TGF-beta and downregulation of BMP signaling. These perturbations disturb growth plate morphogenesis and ultimately lead to the abnormal development of bones formed by endochondral ossification, resulting in abnormal development of the feet after E15 and small stature. This appears consistent with the observed human skeletal phenotype, however, as the sisters were adults, there are no data available to demonstrate whether the human developmental mechanism is the same.

A possible functional role for *CHST11* in lymphocyte development and/or differentiation is less well understood. In adult human tissues, *CHST11* expression is enriched in hematopoietic cells including peripheral leukocytes, the thymus, lymph nodes, and the spleen (BioGPS; Hiraoka et al. 2000). During the late stages of murine embryogenesis (E15), *CHST11* is expressed in a punctate fashion in the liver, suggestive of expression in embryonic hematopoietic cells (Klüppel et al. 2002). Notably, the *CHST11−/−* mouse model was not reported to develop abnormalities of lymphoid tissue or lymphocyte proliferation or differentiation. However, the early death of *CHST11−/−* mice may have precluded the later development of a lymphoproliferative disorder. Alternatively, a phenotype was present in some form but was not evaluated, given the lack of any prior evidence suggesting a functional role for *CHST11* in lymphocytes. Finally, there may be true differences between the function of *CHST11* in humans and mice.

Nonetheless, prior experimental evidence implicates a role for proteoglycan sulfation in lymphocyte differentiation, supporting a plausible contribution of *CHST11* deficiency to malignant lymphoproliferative disease. The enzymes required for biosynthesis of chondroitin sulfate and heparin sulfate appear to be developmentally regulated in B-cell progenitors, resting B cells, and activated B cells (Duchez et al. 2011). Overexpression of the biosynthetic enzyme for heparin sulfate in the B-cell lineage of transgenic mice, which diminished the chondroitin sulfate/heparin sulfate ratio, resulted in a partial differentiation block at the pro-B- to pre-B-cell transition (Duchez et al. 2011). In a two-step culture system, chondroitin sulfate B (CS B) was found to promote IL-4/5 driven murine B-cell differentiation in a dose-dependent fashion, as assayed by IgM production, the number of IgM-secreting cells, and the number of CD138-positive cells (Yoshihara et al. 2007). While similar studies investigating T lymphocyte differentiation do not appear to have been performed, the findings for B lymphocytes support a probable role for chondroitin sulfate in normal lymphocyte differentiation. Notably, chondroitin sulfate was also found to be the major secreted GAG in three leukemic cell lines (Jurkat, a T-cell leukemia model; Daudi, a Burkitt’s or B-cell leukemia model; and THP-1, an acute monocytic leukemia cell line) and the major GAG retained at the membrane in two of the cell lines (Jurkat, Daudi) as assessed by chromatography (Makatsori et al. 2001). Whether chondroitin is abnormally sulfated in these cells is presently unclear.

In addition to impaired differentiation, another hallmark of the transformed phenotype is dysregulated proliferation. Emerging evidence suggests that *CHST11* may regulate cellular proliferation downstream of RAS/MAPK signaling, a pathway dysregulated in both congenital syndromes with risk of malignancy and many sporadic cancers (Rauen 2013). In fibroblasts isolated from a patient with Costello syndrome, a disorder caused by germline activating mutations in *HRAS*, *CHST11* mRNA, and protein expression and chondroitin-4-sulfate were all found to be markedly reduced (Klüppel et al. 2012). Moreover, misexpression of oncogenic HRAS in normal fibroblasts repressed *CHST11* expression, indicating that *CHST11* is a negatively regulated target gene downstream of HRAS signaling. Furthermore, overexpression of wild-type *CHST11* in the *HRAS*-mutant fibroblasts rescued the excessive proliferation associated with HRAS gain of function, demonstrating that restoration of *CHST11* is sufficient to reverse an important cellular phenotype associated with malignancy. It is unknown at the present time whether somatic activating MAPK pathway mutations commonly identified in a range of sporadic malignancies also downregulate *CHST11*, and whether a change in expression of the latter contributes to the malignant phenotype.

Based on these data, we propose the hypothesis that loss of normal chondroitin sulfation may predispose affected individuals to a clonal lymphoproliferative disorder. It is unclear whether manifestation of the phenotype requires the contribution of other germline variants, environmental factors, or somatic genetic mutations in the lymphocyte lineage. It is tempting to speculate that loss of *MIR3922*, the microRNA that lies within the *CHST11* locus at 12q23 and is nullizygous in our proband, contributes to the lymphoproliferative phenotype. Unfortunately, no functional data are available at the present time for this microRNA.

In summary, we have identified *CHST11/MIR3922* deletion as the presumptive cause of the complex phenotype observed in our proband. While *CHST11* deletion alone could account for the patient’s bony malformations, the contribution of the deletion to her T-cell lymphoproliferative disorder is less clear. Concomitant loss of *MIR3922* and perhaps additional events (genetic or environmental) may be required for full expression of the combined phenotype, or it is possible that the genetic etiology of the lymphoproliferative disorder in each sister is distinct. Future studies could aim to directly test whether *CHST11* expression is functionally required for the normal differentiation and/or proliferation of T lymphocytes, using cell lines or other in vitro models of lymphocyte differentiation. Moreover, it would be useful to assess the expression and function of *MIR3922* in lymphocytes, both alone and in combination with reduced expression of *CHST11*. By analyzing mRNA and protein levels, it would also be of interest to assess whether *CHST11* expression is reduced in sporadic hematologic malignancies, particularly because somatic dysregulation at the genetic level was not commonly observed in the Broad TumorPortal or the COSMIC database. Finally, additional evidence linking *CHST11/MIR3922* deletion to our proband’s phenotype may be derived from ascertainment and genetic testing of additional families sharing a similar phenotype.

## Supporting Information

**Table S1.** Primer sequences and transcript information for ddPCR assays. **Table S2.** (A) Results of microsatellite genotyping of fibroblasts and buccal epithelial cells. (B) Chimerism in the buccal epithelia.
