# Supplementary material for: Inherited CHST11/MIR3922 deletion is associated with a novel recessive syndrome presenting with skeletal malformation and malignant lymphoproliferative disease
Source: Mol Genet Genomic Med. 2015 May 10;3(5):413–23. doi: 10.1002/mgg3.152 (PMC4585449; doi:10.1002/mgg3.152)
Supplement: Supplementary file 1 [file mgg30003-0413-sd1.docx]

**Supplementary Table 1**

**Caption: Primer sequences and transcript information for ddPCR assays.**

| **Assay Name** | **MGB probe sequence** | **Forward Primer Sequence** | **Reverse Primer Sequence** | **Label (FAM/VIC)** | **Chr #** | **Transcript ID** |
| --- | --- | --- | --- | --- | --- | --- |
| CHST11.i1_FAM | 5'-CTTGACTTGACTCTTTCCA-3' | 5'-AGCACACCTGTGGGACTTCTG-3' | 5'-GCATGCTTTGAAAACGTCTGACT-3' | FAM | 12 | NM_018413.5 |
| CHST.e2_FAM | 5'-TTCCTCAGTCATGCGGAG-3' | 5'-CATTTCTGATGAGCCTTCACTTTC-3' | 5'-AGATGTCCACACCAAAGGGATT-3' | FAM | 12 | NM_018413.5 |
| RPP30e1_FAM | 5'-CTGACCTGAAGGCTCT-3' | 5'-GATTTGGACCTGCGAGCG-3' | 5'-GCGGCTGTCTCCACAAGT-3' | FAM | 10 | NM_001104546.1 |
| RPP30e1_VIC | 5'-CTGACCTGAAGGCTCT-3' | 5'-GATTTGGACCTGCGAGCG-3' | 5'-GCGGCTGTCTCCACAAGT-3' | VIC | 10 | NM_001104546.1 |
| AP3B1i22_FAM | 5'-TCGCTGACCTTCCCTC-3' | 5'-GCTGAACTGAGCGGAATTGGA-3' | 5'-CCCAGCCTCATCTCTCATACG-3' | VIC | 5 | NM_003664.3 |
| AP3B1i22_VIC | 5'-TCGCTGACCTTCCCTC-3' | 5'-GCTGAACTGAGCGGAATTGGA-3' | 5'-CCCAGCCTCATCTCTCATACG-3' | VIC | 5 | NM_003664.3 |
| NFAT5e1_VIC | 5'-ACGAGGTACCTCAGTGTT-3' | 5'-CATGAGCACCAGTTCCTACAATGAT-3' | 5'-TGCTTTGGATTTCGTTTTCGTGATT-3' | VIC | 16 | NM_138714.2 |

**Supplementary Table 2A**

**Caption: Results of microsatellite genotyping of fibroblasts and buccal epithelial cells.**

| **FIBROBLAST** | | |  | **SALIVA** | | | | |
| --- | --- | --- | --- | --- | --- | --- | --- | --- |
| **Markers** | **1*** | **2*** |  | **Markers** | **1*** | **2*** | **3*** | **4*** |
| D8S1179 | 12 | 13 |  | D8S1179 | 12 | 13 | 10 | 14 |
| D21S11 | 30 | 30 |  | D21S11 | 30 | 30 | 28 |  |
| D7S820 | 11 | 11 |  | D7S820 | 11 | 11 | 10 | 13 |
| CSF1PO | 11 | 12 |  | CSF1PO | 11 | 12 | 10 | 13 |
| D13S317 | 12 | 13 |  | D13S317 | 12 | 13 | 11 |  |
| D2S1338 | 17 | 19 |  | D2S1338 | 17 | 19 | 20 |  |
| D19S433 | 14 | 15 |  | D19S433 | 14 | 15 | 13 | 15.2 |
| vWA | 17 | 18 |  | vWA | 17 | 18 | 15 |  |
| TPOX | 8 | 11 |  | TPOX | 8 | 11 | 9 |  |
| D18S51 | 12 | 14 |  | D18S51 | 12 | 14 | 10 | 15 |
| Amelogenin | X | X |  | Amelogenin | X | X | Y |  |
| D5S818 | 11 | 11 |  | D5S818 | 11 | 11 | 12 |  |
| FGA | 20 | 21 |  | FGA | 20 | 21 | 25 |  |
| D3S1358 | 16 | 18 |  | D3S1358 | 16 | 18 |  |  |
| TH01 | 8 | 9 |  | TH01 | 8 | 9 |  |  |
| D16S539 | 11 | 12 |  | D16S539 | 11 | 12 |  |  |

**Supplementary Table 2B**

**Caption: Chimerism in the buccal epithelia.**

| **Marker** | **Chimerism** |
| --- | --- |
| D8S1179 | 0.62 |
| D7S820 | 0.62 |
| CSF1PO | 0.61 |
| D13S317 | 0.67 |
| D2S1338 | 0.57 |
| vWA | 0.65 |
| TPOX | 0.62 |
| D18S51 | 0.67 |
| D5S818 | 0.59 |
| FGA | 0.58 |
| **Mean %** | **0.62** |
| **SD** | 0.03 |
